# Computed tomography-based assessment of radiographic progression in spine and sacroiliac joints after pregnancy in women with radiographic axial spondyloarthritis

**DOI:** 10.3389/fmed.2022.970546

**Published:** 2022-12-15

**Authors:** Kyung-Ann Lee, So Yun Lee, Se Hee Kim, Hyun-Sook Kim, Hae-Rim Kim, Sang-Hoon Lee

**Affiliations:** ^1^Division of Rheumatology, Department of Internal Medicine, Soonchunhyang University Seoul Hospital, School of Medicine, Soonchunhyang University, Seoul, Republic of Korea; ^2^Department of Rheumatology, Kyung Hee University Hospital at Gangdong, College of Medicine, Kyung Hee University, Seoul, Republic of Korea; ^3^Division of Rheumatology, Department of Internal Medicine, Konkuk University Medical Center, Research Institute of Medical Science, School of Medicine, Konkuk University, Seoul, Republic of Korea

**Keywords:** axial spondyloarthritis, radiographic progression, pregnancy, computed tomography, spine, sacroiliac joint

## Abstract

**Background:**

Mechanical stress are one of the pathogenesis of axial spondyloarthritis (axSpA). During pregnancy, the mechanical overload on the spine and pelvis increases due to gravid uterus. We aimed to investigate whether pregnancy affects radiographic progression in patients with radiographic axSpA (r-axSpA) based on computed tomography (CT) evaluations.

**Materials and methods:**

This retrospective study included women with r-axSpA aged 19–49 years who underwent at least two CT evaluations of the whole spine and/or sacroiliac joints (SIJs) at intervals of 2–4 years. To compare radiographic progression after delivery, we classified the patients into two groups: delivery group and controls. The delivery group was restricted to women who had the first CT ∼2 years before delivery and the second CT ∼2 years after delivery. The CT Syndesmophyte Score (CTSS) (0–522) and SIJ scores (0–40) were used to evaluate spinal syndesmophytes and erosion, joint space narrowing, and sclerosis of the SIJs.

**Results:**

A total of 21 women in the delivery group and 38 women in the control group were included. The median (Q1–Q3) CTSS at baseline in the delivery group and controls was 19 (16–23) and 20 (13.25–27.75), and the median progression was 1 (0–3) and 0 (0–1) during the median 2.9-year follow-up, respectively. The median (Q1–Q3) SIJ score at baseline in the delivery group and controls was 13 (8–22) and 11 (6–22), and the median progression was 1.5 (0–3) and 1 (0–2), respectively. Using cut-off 0.5, 52.9, and 61.9% of r-axSpA patients and 39.3 and 44.4% of controls showed progression of whole spine and SIJs, respectively. However, no difference in proportion of spinal and SIJ progression and absolute score changes per time point was observed between two groups. Moreover, the SIJ score changes were comparable according to the delivery method.

**Conclusion:**

Pregnancy and delivery do not affect the radiographic progression of the spine and SIJs in women with r-axSpA assessed by CT.

## 1 Introduction

Axial spondyloarthritis (axSpA) is a chronic inflammatory arthritis affecting the sacroiliac joints (SIJs) and the spine, commonly occurring in childbearing age (1). It was previously thought that axSpA mainly affected men, but recent studies have shown that the ratio of axSpA in men to women is 3:1 (2). Pregnancy is a major issue faced by female axSpA patients of childbearing age.

Some studies suggest that mechanical stress contributes to axSpA (3). During pregnancy, the combination of mechanical and hormonal changes causes excessive load on the spine and pelvis and alterations of the pelvic ligaments, leading to pelvic and low back pain (4). During pregnancy and after childbirth, almost half of pregnant women experience pain in the lumbar and/or sacroiliac area and/or in the symphysis pubis (5). More women with axSpA discontinue medications during pregnancy than women with systemic lupus erythematosus and rheumatoid arthritis (6), and tumor necrosis factor inhibitor (TNFI) discontinuation in early pregnancy has been identified as a risk factor for disease flares during pregnancy (7). Previous studies on the effects of pregnancy in patients with axSpA have focused on disease activity during pregnancy, pregnancy outcomes [e.g., rates of cesarean section (CS), preterm birth, and preeclampsia], and fetal outcomes (e.g., low birth weight and congenital abnormalities) (7–9). However, the possible influence of biomechanical stress and discontinuation of treatment during pregnancy on radiographic progression in patients with axSpA has not yet been documented.

Recently, the Computed Tomography (CT) Syndesmophyte Score (CTSS) was developed to analyze spinal damage in patients with AS (10). Although the modified Stoke Ankylosing Spondylitis Spine Score (mSASSS) includes the evaluation of lateral views of the cervical and lumbar spine segments, the CTSS has the advantage of evaluating bone proliferation in the entire spine, including the thoracic spine, at four quadrants (anterior, posterior, left, and right) with excellent reliability (11, 12). With respect to imaging evaluation of the SIJs, CT has higher sensitivity and reliability than conventional radiography in detecting sacroiliitis (13, 14).

In this study, we aimed to investigate whether pregnancy and delivery affect the radiographic progression of the spine and SIJs in women with radiographic axSpA (r-axSpA) based on CT evaluations.

## 2 Materials and methods

### 2.1 Study design and population

This retrospective study included women aged 19–49 years who were diagnosed with r-axSpA and fulfilled with the modified New York criteria (15) at Gangdong Kyung Hee University Hospital and Konkuk University Medical Center, South Korea, between February 2009 and August 2020. To be included in this study, patients should have undergone at least two CT evaluations of the whole spine and/or SIJs at intervals of 2–4 years.

To compare radiographic progression after delivery, we classified the patients with r-axSpA into two groups: delivery group and control group. The delivery group was restricted to women who had the first CT evaluation at a maximum of 2 years before delivery and the second CT evaluation at a maximum of 2 years after delivery. Controls were defined as women who had two CT evaluations with 2–4-year intervals. Demographic characteristics (age and smoking), clinical data [disease duration, extra-articular manifestations and Bath Ankylosing Spondylitis Disease Activity Index (BASDAI)], method of delivery [vaginal delivery (VD) or CS], laboratory data [human leukocyte antigen-B27 (HLA-B27), C-reactive protein (CRP), and time-averaged CRP], and treatments were collected from the complete medical records. Time-averaged CRP was defined as the mean CRP level during the CT intervals (16). The normal range of CRP was defined as 0–0.5 mg/dL. CRP elevations related to infections or medical interventions, based on a retrospective chart review, were excluded from the analysis. The treatment strategies used were classified as continuous, on-demand, or discontinued treatment.

### 2.2 CT scoring system

The CTSS, ranging from 0 to 552, was used to compare the radiographic progression of the whole spine (10). The anterior and posterior quadrants from the bottom half of C2 to the top half of S1 were scored in the coronal and sagittal planes [23 vertebral units (VUs) in total], scoring eight quadrants per VU. The score reflects the height of a syndesmophyte relative to the intervertebral disc space (IDS). For every quadrant, the score was 0 if no syndesmophyte was present, 1 if a syndesmophyte was present but did not reach 50% of the IDS, 2 if the syndesmophyte reached or crossed 50% of the IDS, and 3 if the syndesmophyte bridged the IDS.

A total of 24 regions of the SIJs were scored in the SIMACT study (14). However, the present study utilized preexisting CT scans, images were not reformatted into an oblique coronal view oriented parallel and perpendicular to the long axis of the sacrum. Therefore, we used modified scoring system for SIJs. The SIJs were divided into the left and right segments and the iliac and sacral segments, for a total of four segments. The scoring of the radiographs considered erosions, joint space alterations, and sclerosis. Erosions were graded from 0 to 3 [grade 0, no erosions; grade 1, small, isolated erosions (1–2) or a questionable single erosion; grade 2, definite erosions (3–5; < 3 mm) or a larger single erosion (> 3 mm); and grade 3, multiple (> 5) or confluent erosions]. By adding the number of erosions in all four segments in both transverse and coronal views, the total erosion score (TES) was calculated (0–24). The joint space alterations were graded from 0 to 4 (grade 0, no joint space changes; grade 1, questionable widening or narrowing; grade 2, pseudo-widening; grade 3, partial ankyloses; and grade 4, extensive/total ankyloses). Ankylosis was defined as > 1 cm of marrow contiguous between the sacral and iliac sides. Extensive ankylosis was defined as affecting at least half of the joints. Sclerosis was graded from 0 to 2 [grade 0, no sclerosis; grade 1, questionable or little sclerosis (≥ 5 mm); and grade 2, evident sclerosis (≥ 10 mm)]. The total SIJ score ranged from 0 to 40.

The CT scans were independently scored by two trained readers (K-AL and SL). The readers received training including a teaching session and consensus scoring of test cases from a senior rheumatologist (S-HL) with special expertise in SpA prior to beginning the scoring. To avoid reader bias, the readers were blinded to patient characteristics, clinical data, findings of other imaging modalities, and time point of CT scans.

### 2.3 CT scanning technique

The CT scans and radiographs were read using a Digital Imaging and Communications in Medicine viewer. CT evaluations were performed with contiguous slices of 2-mm thickness. All spinal CT scans included a complete vertebral column. Because this study utilized preexisting scans, radiation exposure was not controlled and images of the SIJs were not reformatted into a semicoronal view.

### 2.4 Statistical analysis

Statistical analyses were performed using SPSS for Windows (version 22.0; SPSS Inc., Chicago, IL, USA). Continuous variables are expressed as medians (Q1–Q3), and categorical variables are presented as frequencies and proportions. The results were compared using Pearson’s chi-square test, Fisher’s exact test, and the Mann-Whitney *U* test or Kruskal-Wallis test, as appropriate, for significant differences in patient characteristics between the delivery group and the control group. Interreader reliability was assessed using the intraclass correlation coefficient (ICC). The CT scores between the two time points were compared using the paired Wilcoxon test. The average score was used for the analysis. The SDC is the smallest change that can be detected in an individual patient beyond measurement error and was calculated by SDC = 1.96*SDdiff/(√k*√2). SDdiff is the SD of the difference in raw progression scores between two readers; k is the number of readers (10). Statistical significance was set at *p* < 0.05.

## 3 Results

### 3.1 Baseline characteristics

A total of 45 women with r-axSpA had both spine and pelvic CT evaluations at baseline and follow-up (minimum 2 years, maximum 4 years): delivery group (*n* = 17) and controls (*n* = 28). Fifteen women with r-axSpA had only pelvic CT at baseline and follow-up: delivery group (*n* = 4) and controls (*n* = 10).

Table 1 summarizes the baseline characteristics of the study population. The median (Q1–Q3) age of the r-axSpA patients in the delivery and control groups was 32 (30–34.25) and 30 (26–34.25) years, with a median (Q1-Q3) disease duration of 3 (0.87–6.0) and 1 (0.17–3.0) years, respectively. The median (Q1–Q3) interval time between the first and second CT in the delivery and control groups was 2.96 (2.33–3.47) and 2.95 (2.43–3.33) years, respectively.

**TABLE 1:** Baseline characteristics of women with r-axSpA included in the analyses.

	Delivery (*n* = 21)	Controls (*n* = 38)	*P*-value
**Demographic variables**			
Age at baseline CT, years	32 (30, 34.25)	30 (26, 34.25)	0.072
Smoking	0 (0)	1 (2.6)	1.000
Interval time between the 1st and 2nd CT, years	2.96 (2.33, 3.47)	2.95 (2.43, 3.33)	0.818
Interval time between the delivery and 2nd CT, years	1.12 (0.41, 1.53)		
BMI at baseline CT, kg/m^2^	21.7 (2.5)	21.8 (3.0)	0.917
BMI at follow-up CT, kg/m^2^	21.6 (2.7)	21.6 (2.9)	0.961
**Disease-related variables**			
Disease duration, years	3 (0.87–6.0)	1 (0.17–3.0)	0.083
HLA-B27 positive	20 (95.2)	33 (86.8)	0.407
History of uveitis	7 (33.3)	14 (36.8)	0.694
History of psoriasis	0 (0)	1 (2.6)	1.000
History of IBD	0 (0)	1 (2.6)	1.000
History of enthesitis	6 (28.6)	8 (21.1)	0.583
History of peripheral arthritis	5 (23.8)	11 (28.9)	0.600
BASDAI at baseline CT	3.8 (2.3)	2.9 (2.1)	0.189
BASDAI at follow-up CT	2.48 (1.46)	1.8 (1.0)	0.069
CRP at baseline CT, mg/dl	0.32 (0.08, 0.69)	0.27 (0.1, 0.46)	0.079
CRP at baseline CT > 0.5 mg/dl, n (%)	8 (38.1)	7 (18.4)	0.122
CRP at follow-up CT, mg/dl	0.11 (0.07, 0.75)	0.05 (0.04, 0.30)	0.034
CRP at follow-up CT > 0.5 mg/dl, n (%)	6 (28.6)	3 (7.9)	0.063
Time-average CRP during follow-up, mg/dl	0.36 (0.25, 0.57)	0.17 (0.12, 0.34)	0.006
Time-average CRP during follow-up > 0.5 mg/dl, n (%)	7 (33.3)	6 (15.8)	0.197
Treatment during follow-up			<0.001
Continuous treatment	1 (4.8)	30 (78.9)	
On demand treatment	1 (4.8)	1 (2.6)	
Discontinued treatment	19 (90.5)	7 (18.4)	
Stop duration in patients who discontinued treatment, months	12 (9.5, 24.0)	14 (10.5, 18)	0.760
**Medication during follow-up**			
NSAID/Cox-2 inhibitor	13 (61.9)	32 (84.2)	0.030
csDMARD	7 (33.3)	12 (31.6)	0.985
TNF-alpha blocker	2 (9.5)	9 (23.7)	0.299
IL-17 inhibitor	0 (0)	1 (2.6)	1.000

Results are presented in median (Q1, Q3) or number (%).

r-axSpA, radiographic axial spondyloarthritis; CT, computed tomography; BMI, body mass index; HLA-B27, human leucocyte antigen B27; IBD, inflammatory bowel disease; BASDAI, Bath Ankylosing Spondylitis Disease Activity Index; CRP, C-reactive protein; NSAID, non-steroidal anti-inflammatory drugs; Cox, cyclooxygenase; csDMARD, conventional synthetic disease modifying antirheumatic drug; TNF, tumor necrosis factor; IL, interleukin.

No apparent differences were observed between the two groups in terms of HLA-B27 positivity, extra-articular manifestations, CRP levels at baseline CT, medications, and smoking status. The median CRP levels at follow-up CT and the time-average CRP levels were higher in the delivery group than in controls [0.11 vs. 0.05 mg/dL (*p* = 0.034) and 0.36 vs. 0.17 mg/dL (*p* = 0.006), respectively]; however, the levels were within the normal range in both groups. In the delivery group, 19 women (90.5%) discontinued r-axSpA medications for a median (Q1–Q3) of 12 (9.5–24) months because of pregnancy and/or breastfeeding (*n* = 17) and inactive disease (*n* = 2). In the control group, seven women discontinued treatment during the follow-up period because of inactive disease. The median (Q1–Q3) duration of treatment discontinuation was 14 (10.5–18) months.

### 3.2 Changes in CT scores of the spine over time

Table 2 shows the median status scores per time point and the change scores for the spine and SIJs. The median (Q1–Q3) baseline CTSS for the whole spine in the delivery group and controls was 19 (16–23) and 20 (13.25–27.75), respectively (*p* = 0.622). The median (Q1–Q3) change score of delivery and control groups were 1 (0–3), and 0 (0–1), respectively. The total CTSS significantly increased during the median 2.9 years of follow-up in both groups (*p* < 0.05). Most structural damage and bone proliferation were seen in the thoracic segments. The ICC for the baseline status score was 0.979 for the whole spine and 0.848–0.963 for the spine segments. The ICC for the change scores was 0.926 for the whole spine and 0.848, 0.940, and 0.647 for the cervical, thoracic, and lumbar spine segments, respectively.

**TABLE 2:** Changes in computed tomography (CT) scores over time in r-axSpA women with and without delivery.

	Delivery group	Control group	ICC (95% CI)
	Median (Q1–Q3)	Median (Q1–Q3)	
**Whole spine (0–552)**			
Baseline	19 (16–23)	20 (13.25–27.75)	0.979 (0.963–0.989)
Follow-up	20 (16–24.5)	20 (13.25–28)	0.989 (0.980–0.994)
Change score	1 (0–3)	0 (0–1)	0.926 (0.867–0.959)
**Cervical spine (0–144)**			
Baseline	0 (0–2.5)	0 (0–1.75)	0.848 (0.725–0.916)
Follow-up	0 (0–3)	0 (0–1.75)	0.876 (0.776–0.932)
Change score	0 (0–0)	0 (0–0)	0.848 (0.725–0.916)
**Thoracic spine (0–264)**			
Baseline	17 (14–20.5)	17 (12.25–24.0)	0.958 (0.973–0.992)
Follow-up	18 (14–22)	18 (12.25–24.75)	0.990 (0.982–0.995)
Change score	0 (0–0.5)	0 (0–1)	0.940 (0.892–0.967)
**Lumbar spine (0–144)**			
Baseline	1 (0–2)	0 (0–2)	0.963 (0.933–0.979)
Follow-up	1 (0–2.5)	0.5 (0–2.75)	0.956 (0.921–0.976)
Change score	0 (0–0)	0 (0–1)	0.647 (0.361–0.806)
**Sacroiliac joint**			
**Total score (0–40)**			
Baseline	13 (8–22)	11 (6–22)	0.998 (0.997–0.999)
Follow-up	14 (8.5–20.5)	13 (6–23.5)	0.999 (0.999–1.000)
Change score	1.5 (0–3)	1 (0–2)	0.997 (0.995–0.998)
**Total erosion score (0–24)**			
Baseline	7 (2–14.5)	6.5 (2–14.5)	1.000 (1.000–1.000)
Follow-up	9 (3.5–12.0)	8.5 (3.75–16.5)	1.000 (1.000–1.000)
Change score	1 (0–2)	1 (0–2)	0.994 (0.989–0.996)
**Joint space narrowing (0–8)**			
Baseline	3 (3–4)	3 (0.75–4)	0.996 (0.993–0.998)
Follow-up	4 (3–4)	3.5 (1–4)	0.998 (0.997–0.999)
Change score	0 (0–0)	0 (0–0)	0.997 (0.996–0.999)
**Sclerosis (0–8)**			
Baseline	2 (1–3)	2 (0.75–3)	0.992 (0.987–0.995)
Follow-up	2 (1–3)	2 (0–4)	0.996 (0.993–0.998)
Change score	0 (0–1)	0 (0–0.25)	0.878 (0.801–0.926)

r-axSpA, radiographic axial spondyloarthritis; CT, computed tomography; ICC, intraclass correlation coefficient.

**FIGURE 1:**
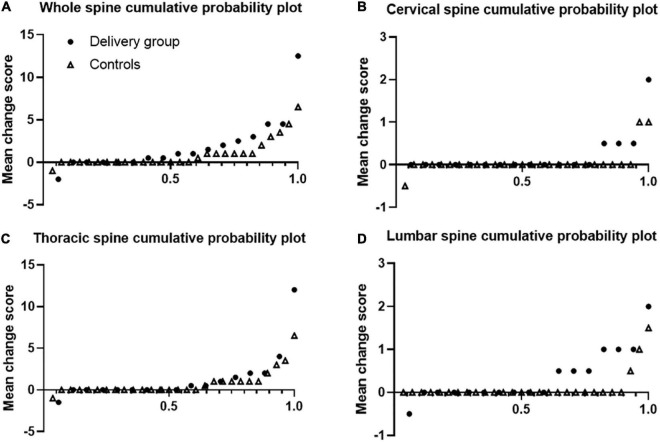
Cumulative probability plots of progression of syndesmophytes. Syndesmophytes were scored with the computed tomography syndesmophyte score (CTSS). Plots are shown for the whole spine **(A)**, cervical **(B)**, thoracic **(C)**, and lumbar **(D)** segments. Dots represents delivery group and triangle represents control group.

Figure 1 shows that r-axSpA women in delivery group showed more progression of whole, cervical and lumbar spine than those in control group. Using 0.5 as cut-off, 52.9% of r-axSpA patients in delivery group and 39.3% of patients in control group had whole spine progression over median 3 year follow-up (Table 3). When using the SDC (1.3) as cut off, 41.1% of r-axSpA women in delivery group and 17.9% of those in control group had 3-year whole spine progression. However, a significant difference in the progression of whole spine and each segments was not observed between the two groups.

**TABLE 3:** Number of r-axSpA women with progression of spine and sacroiliac joints over median 3-year follow-up according to delivery.

	Delivery (*n* = 17)	Control (*n* = 28)	*P*-value
**Whole spine**			
Change > 0.5	9 (52.9)	11 (39.3)	0.371
Change > SDC (1.3)	7 (41.2)	5 (17.8)	0.163
**Cervical spine**			
Change > 0.5	1 (5.8)	2 (7.1)	1.000
Change > SDC (0.3)	4 (23.5)	2 (7.1)	0.179
**Thoracic spine**			
Change > 0.5	6 (35.3)	10 (35.7)	0.219
Change > SDC (1.1)	5 (29.4)	4 (14.3)	0.609
**Lumbar spine**			
Change > 0.5	4 (23.5)	2 (7.1)	0.179
Change > SDC (0.6)	4 (23.5)	2 (7.1)	0.179

**Sacroiliac joints**	**Delivery (*n* = 21)**	**Control (*n* = 36)**	***P*-value**

**Total score**			
Change > 0.5	13 (61.9)	16 (44.4)	0.203
Change > SDC (0.3)	13 (61.9)	17 (47.2)	0.284
**Erosion**			
Change > 0.5	6 (28.6)	8/34 (23.5)	0.677
Change > SDC (0.1)	6 (28.6)	8/34 (22.2)	0.677
**Joint space narrowing**			
Change > 0.5	5 (23.8)	4 (11.1)	0.266
Change > SDC (0.2)	5 (23.8)	6 (16.7)	0.511
**Sclerosis**			
Change > 0.5	9 (42.9)	9/34 (26.5)	0.284
Change > SDC (0.2)	11 (52.4)	13/34 (38.2)	0.304

Results are presented in number (%).

r-axSpA, radiographic axial spondyloarthritis; SDC, smallest detectable change.

### 3.3 Changes in CT scores of the SIJs over time

The median (Q1–Q3) SIJ score at baseline in the delivery group and controls was 13 (8–22) and 11 (6–22), and the median progression was 1.5 (0–3) and 1 (0–2), respectively (Table 2). The total SIJ score significantly increased during the median 2.9 years of follow-up in both groups (*p* < 0.05). Very good reproducibility was observed in the total score (0.997–0.999), TES (0.994–1.000), joint space narrowing (JSN) score (0.996–0.998), and sclerosis score (0.878–0.992) of the SIJs.

**FIGURE 2:**
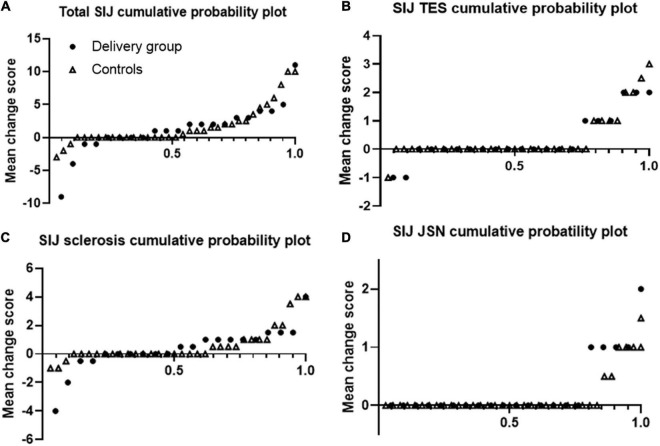
Cumulative probability plots of progression of sacroiliac joints. Plots are shown for the total **(A)** erosion **(B)**, sclerosis **(C)**, and joint space narrowing **(D)** scores. Dots represents delivery group and triangle represents control group.

Figure 2 shows that over half of the patients had SIJ progression over median 3 year-follow up. Using cut-off 0.5, 61.9% of r-axSpA patients in delivery group and 44.4% of patients in control group had SIJ progression (Table 3). In regard to progression (> 0.5) of joint space narrowing and sclerosis, delivery group (23.8 and 42.9%) had more progression than control groups (11.1 and 26.5%) without statistical difference. No differences in the absolute changes in the total, erosion, JSN, and sclerosis scores of the SIJs per time point were observed between the delivery group and controls.

We analyzed the interval changes of SIJs according to the delivery method [CS (*n* = 9) vs. VD (*n* = 3)] in the delivery group (data on the delivery method were missing for nine patients). The changes in the total SIJ score, TES, and JSN and sclerosis scores were comparable between the CS and VD groups.

## 4 Discussion

The present study assessed, using CT, the radiographic progression of the spine and SIJs in women with r-axSpA with and without delivery. We found that the interval changes in the CT scores of the spine and SIJs were not significantly different between r-axSpA patients with and without delivery. The interaction between biomechanical stress and the innate immune response has been proposed as a cause of r-axSpA. According to animal models, mechanical strain can contribute to entheseal inflammation and new bone formation (3). During pregnancy, the combination of mechanical and hormonal changes causes excessive load on the spine and pelvis and alterations of the pelvic ligaments, leading to pelvic and low back pain (4). Forces induced by gravid uterus can cause hyperlordosis, and the pelvis tilts forward as pregnancy progresses. The SIJs resist this forward rotation. In addition, pregnancy-related hormones cause elasticity of the SIJs. These factors contribute to increasing mechanical strain on the SIJs, and low back (17). Pregnancy also influences the connective tissue composition and bone alignment changes around the pelvis (18). Ursin et al. reported that 70% of pregnant women with axSpA in the third trimester did not take any medication. Discontinuation of TNFIs has been reported to be associated with disease flares in parous women with r-axSpA (9). Therefore, we hypothesized that biomechanical stress and discontinuation of treatment could affect the radiographic progression of the SIJs and spine in patients with AS during pregnancy. However, our study results showed no association between childbirth and structural damage in women with r-axSpA.

A major advantage of using CT for the assessment of syndesmophytes is that CT visualizes the whole spine in the sagittal and coronal axes with many slices. CT imaging studies have shown that more extensive syndesmophytes and more progression occur in the thoracic spine than in the cervical or lumbar spine (19). A recent MRI study also reported a significant involvement of the thoracic spine (in terms of enthesitis and bone marrow edema) in patients with early axSpA (20). However, imaging of the thoracic segment has instead not yet been taken into consideration in evaluation of structural damage in axSpA (21–23). Indeed, the CTSS is a more detailed score for syndesmophyte growth, indicating a height reaching > 50 or < 50% of the IDS, than the mSASSS, which indicates only the presence or absence of syndesmophytes (10). Radiographic assessment of spinal progression in AS has been reported to have limited reliability (12). However, we found that the CT scores had excellent interobserver reliability, consistent with a previous CTSS study (10). With respect to SIJs, conventional radiography underestimated sacroiliitis compared with CT in the SIMACT (SacroIliac Magnetic resonance CT) study (14). Although CT provides complete visualization of structural damage, concerns about radiation exposure have traditionally limited its use in AS patients. However, the development of low-dose CT has led to a reduction in radiation dose and noise. The effective dose in low-dose CT of the whole spine was estimated to be approximately 4 mSv, which is still higher than that of radiography (4 mSv: total dose from cervical, lumbar, and pelvic radiography) (10). Diekhoff et al. showed that the mean radiation exposure with low-dose CT of the SIJs was similar to that with conventional radiography (0.51 vs. 0.52 mSv), with a higher detection rate for sacroiliitis (14). Indeed, an oblique scanning method for the SIJs could avoid radiation exposure to the ovaries and require fewer slices to cover the whole SIJs compared with axial scanning (24). Improvement in software together with the ability to reduce the radiation dose could make CT a feasible method for the evaluation of structural damage in the spine and SIJs in the near future.

Spinal progression was found to be associated with the presence of baseline syndesmophytes, male sex, old age, smoking, high disease activity at baseline, and time-averaged CRP levels (25, 26). An observational 2-year follow-up study on patients with r-axSpA using data from the SIAS (Sensitive Imaging in Ankylosing Spondylitis) study showed a mean (standard deviation [SD]) CTSS change score of 17.9 (19). In our study, the median (Q1–Q3) CTSS change in the delivery group and controls was 1 (0–3) and 0 (0–1), respectively, during a similar interval. In the SIAS study, 84% of the enrolled patients were men with a mean (SD) age of 50 (9.8) years, and 38% of the patients had elevated CRP levels at baseline and higher CTSS [whole spine mean (SD) score: 163 (126)]. However, in our study, all patients with r-axSpA were women of a young age with a much lower CTSS at baseline [median (Q1-Q3) in the delivery group and controls: 19 (16–23) and 20 (13.25–27.75), respectively]. Indeed, the time-averaged CRP levels were within the normal range and disease activity at baseline and follow-up CT was low (BASDAI < 4) in both groups. The discrepancy in baseline characteristics could explain the different results of CTSS changes.

In a previous study, ∼60% of women presenting with postpartum back pain without axSpA had positive magnetic resonance imaging (MRI) findings for sacroiliitis according to the Assessment of SpondyloArthritis International Society definition (27). Eshed et al. also found that bone marrow edema (46%) and subchondral sclerosis (26%) were prevalent peripartum MRI findings (28). However, we could not find a link between these inflammatory changes in the peripartum period and the structural progression of SIJs. Although conventional radiography has limitations in evaluating the SIJs owing to their irregular outline and obliquity, CT is a sensitive method for detecting erosions, bone sclerosis, and ankylosis, which also represent the reference standards for the detection of bony alterations in r-axSpA (29). Garagiola et al. analyzed the pelvic CT scans made within 24 h of an uncomplicated term vaginal delivery in 14 women, showed that widening of the SIJs and gas in the SIJs in one (7%) of the postpartum women compared with none of the control subjects (30). Although radiographic progression of the SIJs was detected by CT in both r-axSpA patients with and without delivery, no significant differences in the interval changes of total, erosion, sclerosis, and JSN scores of the SIJs were found in our study. We expected that women with r-axSpA would have increased sclerosis in the SIJs after delivery, such as osteitis condensans ilii (31). Progression of SIJ sclerosis were prevalent in delivery group than control. However, the statistical significance was not found. We used semiquantitative scores to evaluate the structural damage of the SIJs. Further studies with detailed quantitative measurements, such as the number and size of erosions or the exact thickness of sclerosis, are needed to confirm our study results.

Our study suggested that the delivery method (CS or VD) does not affect the radiographic progression of SIJs in patients with r-axSpA. A recent MRI study showed that MRI scores including Spondyloarthritis Research Consortium of Canada (SPARCC) MRI index, SPARCC MRI structural scores, and sclerosis score were not different between women who gave birth by VD and those who gave birth by cesarean delivery (32). A recent study using a medical claims database also showed that changes in prescription did not significantly differ between CS and VD (33). In the present study, the rate of CS (*n* = 9, 75%) was much higher than that of VD (*n* = 3, 25%) in women with AS. However, the small sample size, especially in the VD group, limited the assessment of structural changes of the SIJs according to the delivery method.

The effect of non-steroidal anti-inflammatory drugs (NSAIDs) on the inhibition of radiographic progression in r-axSpA is controversial. A randomized controlled trial of diclofenac failed to show that this NSAID can prevent structural progression in r-axSpA (34). A *post hoc* analysis of the celecoxib trial identified that r-axSpA patients with elevated acute-phase reactants benefited from treatment with celecoxib with respect to radiographic progression (35). In our study, most r-axSpA patients in the delivery group stopped their medications after the confirmation of pregnancy. NSAIDs were the most prescribed medications, and the proportion of patients taking TNFIs was small in both groups. Indeed, the median time-averaged CRP levels were within the normal range in both groups. Owing to the low disease activity and low use of TNFIs in our study population, treatment discontinuation in pregnancy could not make a significant difference in structural progression between the two groups. In literature, few studies had analyzed the ability of the TNFI and IL-17 inhibitors on radiographic progression with the trend of development/progressive growth of syndesmophytes on spine and worsening of structural damage on SIJ (36, 37). Although the sample size was small, we additionally compared the radiographic progression of spine and SIJs in non-pregnant patients with axSpA with TNFI and those without treatment of TNFI. However, the changes in the CTSS and total SIJ scores were comparable (data not shown). In the future, low dose CT may test treatment effect on structural progression by decreasing the time needed to observe a treatment effect.

Our study had several limitations. First, the statistical power was limited by the small sample size. The small number of patients with at least two CT evaluations in 2–4-year intervals, the relatively low prevalence of r-axSpA in female patients. Inadequate sample size could result in non-positive results in our study. To our knowledge, our study firstly provided insights into the association of pregnancy and delivery with radiographic progression in AS. Therefore, we could not provide evidence by previous literature or analogous literature that results may be otherwise. Our study results should be confirmed in larger studies. Second, the presented study only evaluated structural abnormalities, not inflammatory changes. This study could not include the research of possible correlation of low dose CT detected syndesmophyte with different imaging technique [MRI detected (vertebral corner inflammation and vertebral corner fat deposition)]. A recent study showed that vertebral corner inflammation and vertebral corner fat deposition were positively associated with syndesmophyte development. However, almost half of bone formation occurred in corners without vertebral corner inflammation/fat deposition, suggesting the presence of these MRI lesions does not fully clarify the development of syndesmophytes, and low dose CT can be a useful tool for monitoring bone formation of whole spine in research (38). Third, the retrospective design of this study is a shortcoming. Certain clinical parameters, such as Ankylosing Spondylitis Disease Activity Score and body weight during pregnancy, could not be evaluated through retrospective chart reviews. Information on the delivery method was missing for nine patients. Forth, the present study utilized preexisting scans, and the radiation dose of CT was not uniform between the two centers. Images were not reformatted into an oblique coronal view oriented parallel and perpendicular to the long axis of the sacrum. A total of 24 regions of the SIJs were scored in the SIMACT study (14). However, only four segments with coronal and sagittal views were analyzed in our study, which could have led to a low detection of radiographic progression in the SIJs.

In conclusion, the present study found that pregnancy and delivery have no effects on the radiographic progression of the spine and SIJs in female patients with r-axSpA based on CT scoring methods.

## Data availability statement

The original contributions presented in this study are included in the article/supplementary material, further inquiries can be directed to the corresponding authors.

## Ethics statement

The studies involving human participants were conducted in accordance with the Declaration of Helsinki and approved by the Institutional Review Boards (IRBs) of Gangdong Kyung Hee University Hospital (IRB No. 2019-08-012) and Konkuk University Medical Center (IRB No. KUH1011006). Written informed consent for participation was not required for this study in accordance with the national legislation and the institutional requirements.

## Author contributions

K-AL, H-RK, and S-HL were involved in study conception and design. K-AL, SL, SK, and H-SK were involved in data acquisition. K-AL and S-HL performed the data analysis and interpretation. H-RK and S-HL had full access to all of the data in the study and takes responsibility for the integrity of the data and the accuracy of the data analysis. All authors were involved in the drafting or critical revision of the article and approved the final version for publication.
