# Corrigendum: Computed tomography-based assessment of radiographic progression in spine and sacroiliac joints after pregnancy in women with radiographic axial spondyloarthritis

**DOI:** 10.3389/fmed.2023.1335470

**Published:** 2023-12-18

**Authors:** Kyung-Ann Lee, So Yun Lee, Se Hee Kim, Hyun-Sook Kim, Hae-Rim Kim, Sang-Hoon Lee

**Affiliations:** ^1^Division of Rheumatology, Department of Internal Medicine, Soonchunhyang University Seoul Hospital, School of Medicine, Soonchunhyang University, Seoul, Republic of Korea; ^2^Department of Rheumatology, Kyung Hee University Hospital at Gangdong, College of Medicine, Kyung Hee University, Seoul, Republic of Korea; ^3^Division of Rheumatology, Department of Internal Medicine, Konkuk University Medical Center, Research Institute of Medical Science, School of Medicine, Konkuk University, Seoul, Republic of Korea

**Keywords:** axial spondyloarthritis, radiographic progression, pregnancy, computed tomography, spine, sacroiliac joint

In the published article, there was an error.

A sentence in the Introduction was incorrectly phrased, which may have led to a misconception that axSpA occurs in one out of three women.

A correction has been made to **Introduction**, Paragraph 1. This sentence previously stated: “Although axSpA was previously believed to predominantly affect men, recent studies have shown that the incidence of axSpA in women is as high as one in every three women (2).”

The corrected sentence appears below:

“It was previously thought that axSpA mainly affected men, but recent studies have shown that the ratio of axSpA in men to women is 3:1 (2).”

The authors apologize for this error and state that this does not change the scientific conclusions of the article in any way. The original article has been updated.

